# Ethnicity predicts long-term depressive symptom patterns in older adults with type 2 diabetes

**DOI:** 10.1016/j.inpsyc.2025.100034

**Published:** 2025-01-24

**Authors:** Yuxia Ouyang, David Manier, Ramit Ravona-Springer, Mery Mamistalov, Dar Gelblum, Anthony Heymann, Joseph Azuri, Laili Soleimani, Ruby Phillips, Mary Sano, Michal Schnaider Beeri, Elizabeth Guerrero-Berroa

**Affiliations:** a Department of Population Health Science and Policy, Icahn School of Medicine at Mount Sinai, New York, NY 10029, USA; b Department of Psychology, Lehman College/City University of New York, Bronx, NY 10468, USA; c The Joseph Sagol Neuroscience Center, Sheba Medical Center, Tel HaShomer, Israel; d Department of Psychiatry, Sheba Medical Center, Tel HaShomer, Israel; e Faculty of Medical & Health Sciences, Tel Aviv University, Tel Aviv, Israel; f Maccabi Health Services, Tel Aviv, Israel; g Department of Psychiatry, Icahn School of Medicine at Mount Sinai, New York, NY 10029, USA; h James J. Peters Veterans Affairs Medical Center, Bronx, NY 10468, USA; i Krieger Klein Alzheimer’s Research Center, Brain Health Institute, Rutgers University, New Brunswick, NJ 08901, USA

**Keywords:** Ethnicity, Depressive symptoms, Depression dimensions, Diabetes, Older adults

## Abstract

**Objectives:**

Depression is a chronic disorder that significantly affects functional decline in older adults, especially those with type 2 diabetes (T2D). Ethnic groups may experience different depression risks and severities, yet the effect of ethnicity on depression trajectories and specific dimensions in older adults with T2D remains largely unexamined. We examined the longitudinal associations of ethnicity with depression and its specific dimensions over time in older Ashkenazi and non-Ashkenazi Jews with T2D.

**Design:**

Generalized estimating equations (GEE) models were employed to analyze the longitudinal associations of ethnicity with numbers of depressive symptoms and specific depression dimension, adjusting for sociodemographics, cognition, T2D characteristics, and cardiovascular risk factors.

**Setting:**

Community-dwelling older adults from the longitudinal Israel Diabetes and Cognitive Decline (IDCD) study.

**Participants:**

902 Ashkenazi and non-Ashkenazi Jews, mean age= 72.3 years.

**Measurements:**

The Geriatric Depression Scale-15 (GDS-15) and its five dimensions: Dysphoric Mood, Withdrawal Apathy-Lack of Vigor, Anxiety, Hopelessness, and Memory Complaint.

**Results:**

At baseline, Ashkenazi Jews reported significantly lower GDS-15 scores compared to non-Ashkenazi Jews. They exhibited lower scores in Dysphoric Mood and Hopelessness dimensions. GEE models confirmed these findings, showing Ashkenazi Jews had significantly lower total GDS-15 scores (β = 0.86, 95 % CI 0.75–0.99; p = 0.03), Dysphoric Mood (β = 0.76 (0.52–0.90], p = 0.006), Hopelessness (β = 0.74 [0.58–0.95], p = 0.017) and lower rates of clinical depression (OR= 0.68 [0.52–0.90], p = 0.006). These data offered no evidence of a difference in trends between the Ashkenazi and non-Ashkenazi groups on depression trajectories.

**Conclusions:**

Ethnicity is associated with the longitudinal trajectories of depression and its specific dimensions in older adults with T2D. Further investigation of contributing factors, including social determinants of health, is essential.

## Introduction

Depression is a disorder that negatively affects functioning and quality of life and increases mortality. Older adults and people with type 2 diabetes (T2D) have higher prevalence of depression [17,22,5], and both conditions, including subclinical depression, are associated with cognitive decline and dementia. Indeed, in T2D, social withdrawal, apathy, and lack of vigor are commonly reported depressive symptoms—also correlated with poor cognitive functioning [33]. Identifying factors contributing to increased depression risk and its specific dimensions in older adults at high depression risk such as T2D can pinpoint areas for targeted clinical intervention to reduce this disorder.

Depression has been shown to vary based on ethnicity or country of origin, especially among older adults [11,19,20]. Compared with White Americans, differing levels of depression among minority/disadvantaged ethnic groups, such as African-Americans and Hispanics, have been repeatedly reported [10,37,7]. Results have also varied based on other sociodemographic factors, such as age and gender [12]. In Israel, one study found higher depression rates among very old Israeli residents originating from the Middle East and North Africa [28]. While non-Ashkenazi Jews generally originate from Sephardic or Middle Eastern/North African roots, non-Ashkenazi Jews generally originate from Sephardic or Middle Eastern/North African roots [2].

Data from hospitalized patients with psychiatric disorders suggest higher rates of affective disorders in Ashkenazi Jews, compared with non-Ashkenazi [15]. However, community and population-based studies have found higher depression rates in non-Ashkenazi Jews [16] and also in very old Arab adults [28]. Yet, one population-based cross-sectional study reported no ethnic group differences after adjusting for sociodemographic factors and chronic medical conditions [12]. This discrepancy in findings can be at least partially explained by differences in research design, diagnostic criteria or instrument used, adjustment or lack thereof for confounders, and demographics. The role of ethnicity in longitudinal trajectories of depression and on its distinct dimensions are lacking. Disentangling the potential effect of ethnicity on depression may further contribute to personalized health management. The proposed study capitalized on the Israel Diabetes and Cognitive Decline (IDCD) study to investigate, in 902 older adults with T2D, the associations of Jewish ethnicity (Ashkenazi vs. non-Ashkenazi) with baseline and longitudinal changes in number of depression symptoms, and with depression dimensions.

## Methods

### Participants

The sample for this study consisted of the 1204 participants from the Israel Diabetes Cognitive Decline (IDCD) study, a community-based longitudinal cohort study, whose overall aim is to identify aspects of T2D that are associated with cognitive decline. IDCD participants were initially 65 years old or older with normal cognitive functioning at baseline [4]. Of the 1204, 902 were included in this study as they had complete data on demographic characteristics (age, sex, education, country of origin), global cognition as measured by the Mini Mental State Exam (MMSE), depression (measured by the GDS-15), T2D-related characteristics (HbA1c; duration of T2D), and cardiovascular risk factors (body mass index, creatinine, total cholesterol, triglycerides, and diastolic and systolic blood pressure). Due to the small sample (n = 6), participants with mixed ethnic backgrounds (i.e., having both Ashkenazi and non-Ashkenazi origins) were excluded from this study. All participants were required to speak Hebrew fluently. Study procedures were approved by the Icahn School of Medicine at Mount Sinai, Sheba Medical Center, and Maccabi Heath Services IRB committees. All participants signed informed consent.

All participants underwent baseline assessments. Follow-up assessments were planned at intervals of approximately 18 months. Of the 902 participants, 752 had a second visit with a median follow-up time of 24 months [IQR: 18–24], 536 had a third visit with a median follow-up time of 48 months [IQR: 48–54], and 50 had a fourth visit with a median follow-up time of 72 months [IQR: 72–72]. We used the data from all participants and all assessments for this analysis. Importantly, the lower numbers at follow-up do not necessarily represent dropouts, but rather that participants have not been seen yet for their next follow-up since this is an ongoing longitudinal study.

Comparisons between participants who dropped out from the study after baseline and those who remained in the study for at least one follow-up visit, showed that the groups did not differ in most baseline characteristics (age, sex, education, T2D duration, GDS score, percent of participants with GDS > 5, total cholesterol levels, triglycerides levels, creatinine levels, diastolic blood pressure, and BMI). The only difference (p = 0.027), but not of clinical significance, was in minimally lower MMSE scores among those who dropped out (Supplemental Table 1).

### Depression outcomes

#### Geriatric Depression Scale, 15-item version (GDS-15).

Depression, as measured by the GDS-15 was one of the outcome variables. It is a self-reported measure of depression in older adults. Users respond in a “Yes/No” format. The GDS-15 was selected from the original 30-item version, GDS-30, based on their high correlation with depressive symptoms in preceding validation studies [18,21,24,27,30]. The GDS-15 can be completed in approximately 5 to 7 min, making it ideal for older adults with comorbidities who are easily fatigued or are limited in their ability to concentrate for longer periods of time. Each item is scored as 1 point, with clinical depression defined as having the cutoff score ≥ 5.

#### GDS-15 dimensions.

Other depression outcomes were the GDS-15 dimensions. We and others have used factor analysis on the GDS-15 to identify five dimensions of depression [3,33,34]: *(1) Dysphoric mood*, items 1, 3, 4, 5, 7, 11, and 15: 1. Are you basically satisfied with your life? 3. Do you feel that your life is empty? 4. Do you often get bored? 5. Are you in good spirits most of the time? 7. Do you feel happy most of the time? 11. Do you think it is wonderful to be alive now? And 15. Do you think that most people are better off than you are? *(2) Withdrawal-Apathy-Lack of Vigor (WAV*), items 2, 9, and 13: 2. Have you dropped many of your activities and interests? 9. Do you prefer to stay at home, rather than going out and doing things? and 13. Do you feel full of energy? *(3) Anxiety*, item 6: 6. Are you afraid that something bad is going to happen to you? *(4) Memory complaint*, item 10: 10. Do you feel that you have more problems with memory than most? *and (5) Hopelessness*, items 8, 12, and 14: 8. Do you often feel helpless? 12. Do you feel worthless the way you are now? 14. Do you feel that your situation is hopeless?

### Definition of ethnicity

All participants self-identified as Jews. They were asked about their and their parents’ and grandparents’ country of origin and categorized as Ashkenazi if both parents were from Central and Eastern Europe, including countries such as Germany, Poland, Russia, Romania, Lithuania, Belarus, Ukraine, and Hungary. Participants whose parents and/or grandparents were from Spain, Portugal, North Africa, the Middle East, and Asian countries such as Yemen were considered non-Ashkenazi. Participants reporting mixed ethnic backgrounds (i.e., having both Ashkenazi and non-Ashkenazi origins) were excluded from this study.

### Statistical analyses

Descriptive data were reported as either N (percent) or mean (standard deviation) for categorical and continuous variables, respectively. Two-sample t tests were used to examine the differences in means between Ashkenazi vs. non-Ashkenazi groups, and Chi-square tests were used to examine the association between categorical variables in the two ethnicity groups.

The generalized estimating equations (GEE) method with logit link for negative binomial regression or for logistic regression was fitted to investigate the longitudinal association between Ashkenazi ethnicity and depression scores or dimensions. The GEE method was used to account for multiple visits from the same subject within the study period. The exchangeable working correlation matrix was used and the empirical standard errors of the parameter estimates were reported based on the sandwich estimators.

Given that GDS score represents the count of Yes/No responses to related questions and that Total GDS scores, Dysphoric mood, WAV, and Hopelessness follow a negative binomial distribution, GEE models with a negative binomial distribution were used to estimate these outcomes. GEE models with binomial distribution were used to estimate the binary outcomes, including Clinical Depression (GDS > 5), Anxiety, and Memory complaint. Group differences are expressed as adjusted odds ratios of developing depression or the two dichotomous depression dimensions. The models analyzed the longitudinal association of ethnicity with GDS scores or dimensions by first examining the interaction of ethnicity with time (in years) and then assessing its main effect with the effect of time being controlled. A significant interaction between the ethnicity with time would suggest that ethnicity effect on GDS scores or dimensions were not constant over time. When the interaction term was not significant, we focused on interpreting the main effect models where the effect of ethnicity would not change over time. Two sets of models were used: model 1, adjusted for demographic variables (age, sex, and education) and baseline global cognitive function (as measured by the MMSE); and model 2, additionally adjusted for cardiovascular risk factors (systolic and diastolic blood pressure, total cholesterol, triglycerides, creatinine, and BMI) and T2D-related characteristics (duration of T2D and HbA1c). A two-sided p value of < 0.05 was determined as statistically significant for all tests. Data was analyzed using SAS 9.4 (SAS Inc, Cary, NC).

## Results

**Fig. 1.**
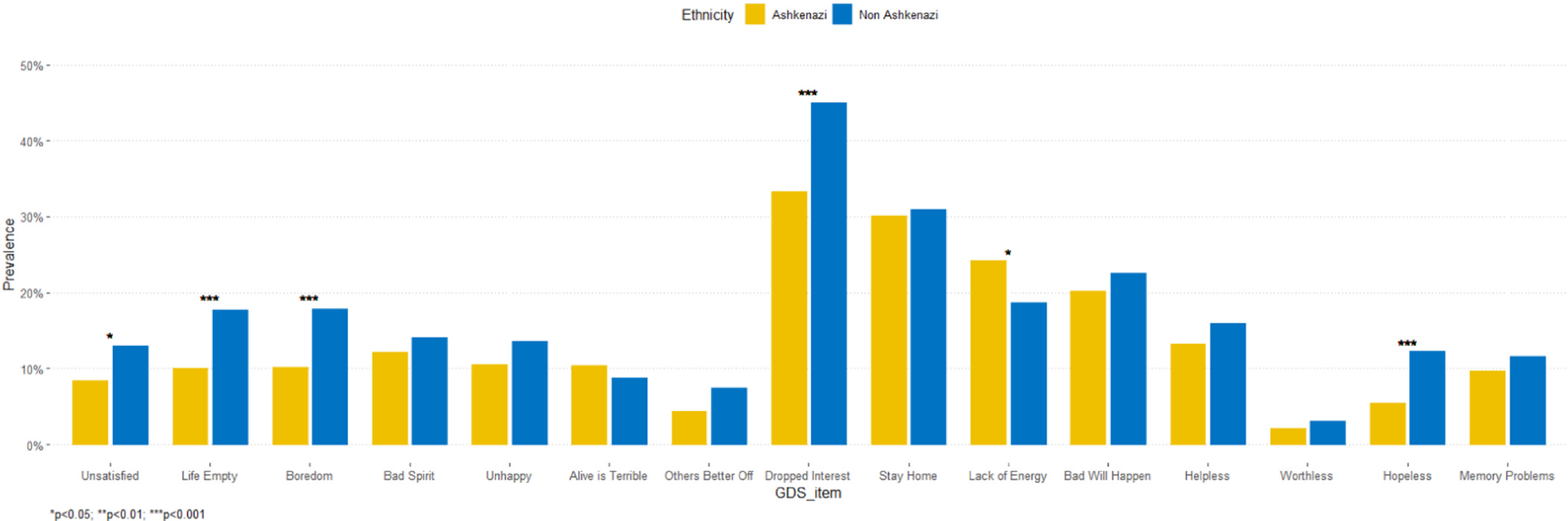
Baseline prevalence of depressive symptoms is higher in non-Ashkenazi compared to Ashkenazi Jews.

The study included 902 participants (463 Ashkenazi, 455 non-Ashkenazi). As shown in Table 1, the two groups did not differ significantly at baseline in age, T2D-related characteristics, and cardiovascular risk factors. Ashkenazi Jews had significantly more years of formal education than non-Ashkenazi Jews (mean = 14.1 [3.46] vs. 12.1 [3.28], p < 0.001) and higher scores on the MMSE (28.4 [1.65] vs. 27.6 [1.84], p < 0.001). Table 2 presents differences in the depression variables in the two ethnicity groups at baseline. The Ashkenazi group had significantly lower GDS-15 total score (Mean [SD] = 2.01 [2.13] vs. 2.48 [2.56], p = 0.003) and lower rate of clinical depression (11.6 % vs. 19.1 %, p = 0.003) than the non-Ashkenazi group. On the GDS-15 dimensions, the Ashkenazi group had significantly lower total mean scores on the Dysphoric Mood (0.095 [0.18] vs. 0.13 [0.22], p = 0.005) and Hopelessness dimension (0.21 [0.51] vs. 0.30 [0.61], p = 0.02) than non-Ashkenazi Jews (Fig. 1).

**Fig. 2.**
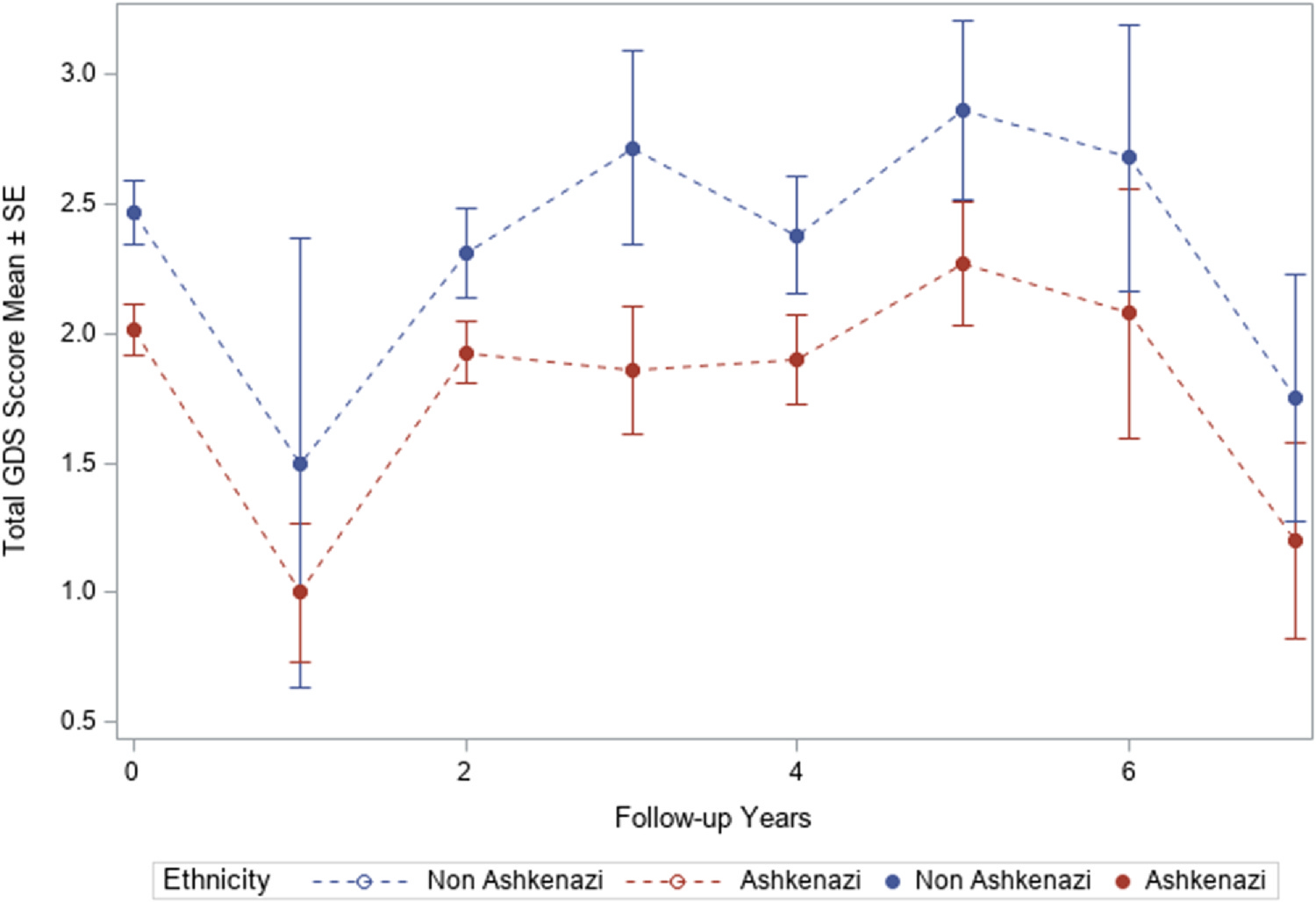
The number of depressive symptoms is consistently higher over time in non-Ashkenazi compared to Ashkenazi Jews.

GEE regression models did not find significant interactions of ethnicity status with time, suggesting that the GDS trajectories for both groups remain parallel over time, with no evidence for significant divergence between the Ashkenazi and non-Ashkenazi groups (see Fig. 2). The main effect models, using model 1, which adjusted for sociodemographics, supported the findings from the bi-variate analysis that the Ashkenazi Jews compared to non-Ashkenazi Jews have significantly lower total GDS-15 scores (Ratio = 0.860, 95 % CI 0.750 – 0.985, p = 0.03), lower Dysphoric Mood scores (Ratio = 0.762, 95 % CI 0.523 – 0.895, p = 0.006), and lower Hopelessness scores (Ratio = 0.738, 95 % CI 0.575 – 0.946, p = 0.017). Ashkenazi Jews were also less likely to be clinically depressed (Odds Ratio = 0.684, 95 % CI 0.523 – 0.895, p = 0.006).

Results remained unchanged for all analyses using model 2, which, in addition to sociodemographic factors, adjusted for cardiovascular risk factors and T2D-related characteristics (Table 3).

## Discussion

Based on a sample of 902 participants, about half of Ashkenazi and half of non-Ashkenazi origins, we examined associations with cross-sectional and longitudinal changes in the number of depression symptoms. Our main finding revealed a significant difference in depression scores between Ashkenazi and non-Ashkenazi participants. At baseline and over time, Ashkenazi individuals consistently scored, on average, lower on the GDS-15 total score and showed lower rates of clinical depression than non-Ashkenazi participants. These differences were constant over time. However, these data offered no evidence of a difference in trends between the two groups on depression status. We also examined which depression dimension may drive the differences between the groups and found that Ashkenazi participants scored lower on the Dysphoric Mood and Hopelessness dimensions; but we did not find evidence of significant differences on the other three dimensions of the depression scale (i.e., Withdrawal-Apathy-Lack of Vigor, Anxiety, and Memory Complaint). Throughout the study, from baseline to all subsequent time points, Ashkenazi individuals consistently demonstrated lower average GDS-15 total scores and lower rates of clinical depression compared to non-Ashkenazi participants. Importantly, while these differences between the groups remained constant, the trajectories of depression over time were parallel, showing no significant divergence between Ashkenazi and non-Ashkenazi individuals. In other words, although Ashkenazi participants maintained lower depression scores at each time point, the pattern of change in depression status over time was similar for both groups.

Non-Ashkenazi older adults have lower socioeconomic (e.g., lower income, poorer quality of education, and neighborhood problems) and psychosocial (e.g., higher chronic stress and less social participation and support) protective factors compared with Ashkenazi Jews [8]. There is evidence that Ashkenazi Jews in Israel have greater access to a range of resources. They tend to have higher income and higher educational achievement, particularly as they get older [6,13]. Although the two groups did not differ in cardiovascular risk factors, less-frequent use of medical care may lead to poorer treatment of such factors, which, in turn, are related to greater risk of depression. In the United States, Blacks/African Americans and Hispanics, who are minority/underserved groups, experience discrimination and are at a greater socioeconomic disadvantage compared to European Whites. These minorities have more chronic and severe depression [25]. Indeed, a common finding has been that, in contrast to Whites, Blacks, and Hispanics have a greater tendency to experience poor social status, low income and education, and poverty—all powerful economic stressors that contribute to a higher prevalence of depressive symptoms [26,35–37].

Abramson et al. have argued that resilience attributes, at both community and individual levels, ranging from social networks to household income, can provide protection against the effects of depression. Income and education may be related to a range of other resilience attributes, from medical care to social networks [2].

Thus, to understand better the nature of the association between ethnicity and depression, it is important to address relevant mechanisms, in particular the social determinants of mental health in older adults. Besides income, education, and social support, social inclusion, stress, housing/living arrangement, physical activity opportunities, age, and gender are also important factors. There is evidence supporting the role of social factors in depression among individuals of different age groups residing in Israel, with minority groups being more affected than Israeli Jews. For instance, associations between economic disadvantage and depressive symptoms were shown in Arab mothers, but not in Israeli mothers [32]. Higher loneliness rates in older Arab adults (65 years and older) compared to Israeli Jews [23] have also been reported. Other contributing factors include gender, physical activities, and acculturation. For example, in older Israelis, especially women, those reporting feeling lonely were less likely to engage in physical activities [23]. Compared to older veteran Jews, older immigrants who arrived in Israel from the former Soviet Union after 1989 were significantly lonelier, but less lonelier over time, suggesting the protective effects of higher acculturation level [9]. Furthermore, immigrant women from Russia and Middle Eastern countries showed more depressive symptoms than immigrant men [38]. Thus, these sociodemographic and sociocultural factors or resilience attributes warrant further investigations to better understand the role of ethnicity in depression and its trajectories.

Overall, our findings showed that ethnicity is related to the specific depression dimensions of Dysphoric Mood and Hopelessness. The lack of evidence of associations between ethnicity and the Memory Complaint and Anxiety dimensions may be partially attributed to their psychometric characteristics, specifically the use of a dichotomous yes/no question rather than a set of symptoms. It is also possible that the lack of evidence for an association between ethnicity and the other depression dimensions, such as Memory Complaint (a depression symptom but also a subjective report of decreased cognitive ability), are more associated with the biological effects of T2D itself. Increased endorsement of the Withdrawal-Apathy-Lack of Vigor dimension has been found to be associated with faster cognitive decline [33] and related brain pathologies. It is noteworthy that depression is a heterogeneous condition not only in terms of its symptomatology, but also in response to treatment and etiology, for which the contribution of a genetic vulnerability potentially associated with these findings cannot be ruled out.

The main limitation of our study is that ethnicity is based on individual self-report and on information about country of origin of parents and grandparents rather than on genetics. Similarly, there was no further distinction within each ethnic group, which can be further categorized based on their distinct cultural values and beliefs. For example, non-Ashkenazi Jews consist of subgroups that include Sephardi and Mizrahi Jews. Although ethnicity, religion or race were not limited in this study, the requirement for fluency in the Hebrew language may have limited the Arab older adult community from participation in this study. Although we have adjusted for numerous sociodemographic variables and directly measured long-term clinical characteristics, we did not have specific information on protective factors/resilience attributes (such as income, social support, and physical activities), which may have differential ethnic effects [29] and could have contributed to our results.

This study has several strengths, including its large sample size, longitudinal design, and the novelty of the associations with specific depression dimensions. It is common knowledge that there are cultural variations in the presentation/expression of depressive symptoms-for example, somatic symptoms such as fatigue and body pain being less stigmatized and in turn more frequently reported in some cultures [14]. These symptoms may also indicate physical ailments and frailty in older adults, particularly those with T2D [1,31], rather than solely representing depressive symptoms. With the exception of lack of energy, the GDS-15 items exclude most somatic symptoms, a strength of this questionnaire in the context of T2D older adults, who often suffer from neuropathic pain.

Depression in its own right, and its association with a myriad of negative health outcomes, has become a major public health concern. Findings from this study highlight the importance of identifying and effectively treating depressive symptoms among non-Ashkenazi Jews with T2D. Addressing the sociodemographic and socioeconomic factors putting these individuals at risk for depression is crucial in research and clinical settings. In the medical setting, healthcare providers should practice culturally sensitive care, by careful attention to nonmedical factors increasing their patients’ risk for depression. Community and religious leaders may be in the position to encourage programs in their communities providing health literacy in general, and specifically about depression and its deleterious effects-especially among groups with low socioeconomic status-in order to foster health-seeking behaviors, healthcare utilization, and lifestyle changes.

## Supplementary Material

1

## Figures and Tables

**Table 1:** Demographic and clinical characteristics of the participants.

	Non-Ashkenazi (N = 446)	Ashkenazi (N = 456)	P-value	Total (N = 902)

**Age (years)**				
Mean (SD)	72.0 (4.52)	72.5 (4.80)	0.06	72.3 (4.67)
**Female sex (%)**	177 (39.7 %)	180 (39.5 %)	1	357 (39.6 %)
**Years of education**				
Mean (SD)	12.1 (3.28)	14.1 (3.46)	< **0.001**	13.1 (3.52)
**Duration of T2D (years)**				
Mean (SD)	9.56 (4.35)	9.88 (4.41)	0.289	9.72 (4.38)
**Cholesterol mg/dl**				
Mean (SD)	175 (25.1)	173 (23.9)	0.236	174 (24.5)
**Creatinine mg/dl**				
Mean (SD)	1.01 (0.339)	1.02 (0.318)	0.774	1.01 (0.328)
**HbA1c (%)**				
Mean (SD)	6.86 (0.829)	6.81 (0.677)	0.338	6.84 (0.757)
**Triglycerides mg/dl**				
Mean (SD)	155 (63.4)	157 (60.2)	0.731	156 (61.8)
**Systolic blood pressure (mmHg)**				
Mean (SD)	135 (8.90)	134 (8.33)	0.224	135 (8.62)
**Diastolic blood pressure (mmHg)**				
Mean (SD)	75.4 (4.69)	75.9 (4.65)	0.181	75.7 (4.67)
**BMI (Kg/m2)**				
Mean (SD)	28.5 (4.03)	28.7 (4.39)	0.587	28.6 (4.21)
**MMSE Total**				
Mean (SD)	27.6 (1.84)	28.4 (1.65)	< **0.001**	28.0 (1.79)

Note: MMSE = Mini-Mental State Examination.

**Table 2:** Descriptive summary for outcome variables by ethnicity at baseline.

	Non-Ashkenazi (N = 446)	Ashkenazi (N = 456)	P-value	Total (N = 902)

**GDS Score**				
Mean (SD)	2.48 (2.56)	2.01 (2.13)	**0.003**	2.24 (2.36)
**Clinical Depression**	85 (19.1 %)	53 (11.6 %)	**0.003**	138 (15.3 %)
**Dysphoric Mood**				
Mean (SD)	0.132 (0.217)	0.0952 (0.181)	**0.005**	0.114 (0.200)
**Unsatisfied (%)**	56 (12.6 %)	36 (7.9 %)	**0.032**	92 (10.2 %)
**Life Empty (%)**	77 (17.3 %)	45 (9.9 %)	**0.002**	122 (13.5 %)
**Boredom (%)**	79 (17.7 %)	47 (10.3 %)	**0.002**	126 (14.0 %)
**Bad Spirit (%)**	61 (13.7 %)	54 (11.8 %)	515	115 (12.7 %)
**Unhappy (%)**	61 (13.7 %)	47 (10.3 %)	0.148	108 (12.0 %)
**Alive is Terrible (%)**	39 (8.7 %)	45 (9.9 %)	0.635	84 (9.3 %)
**Others Better off (%)**	31 (7.0 %)	20 (4.4 %)	0.121	51 (5.7 %)
**WAV**				
Mean (SD)	0.942 (0.867)	0.857 (0.874)	0.147	0.899 (0.871)
**Dropped Interest (%)**	201 (45.1 %)	150 (32.9 %)	< **0.001**	351 (38.9 %)
**Stay Home (%)**	138 (30.9 %)	134 (29.4 %)	0.742	272 (30.2 %)
**Lack of Energy (%)**	81 (18.2 %)	107 (23.5 %)	0.053	188 (20.8 %)
**Anxiety (%)**	101 (22.6 %)	91 (20.0 %)	0.346	192 (21.3 %)
**Bad Will Happen (%)**	101 (22.6 %)	91 (20.0 %)	0.346	192 (21.3 %)
**Hopelessness**				
Mean (SD)	0.298 (0.613)	0.211 (0.509)	**0.02**	0.254 (0.564)
**Helpless (%)**	69 (15.5 %)	61 (13.4 %)	0.423	130 (14.4 %)
**Worthless (%)**	13 (2.9 %)	10 (2.2 %)	0.423	23 (2.5 %)
**Hopelessness (%)**	51 (11.4 %)	25 (5.5 %)	**0.002**	76 (8.4 %)
**Memory Complaint (%)**	50 (11.2 %)	43 (9.4 %)	0.454	93 (10.3 %)

Note: WAV = Withdrawal-Apathy-Lack of Vigor.

**Table 3:** Adjusted associations of ethnicity with longitudinal GDS-15 total score, clinical depression, and depression dimensions.

	Model 1			Model 2		
	
Outcome	Mean Estimate*	95 % CI	*P* value	Mean Estimate*	95 % CI	*P* value

Total GDS Score	0.860	(0.750, 0.985)	**0.030**	0.861	(0.745, 0.996)	**0.043**
Clinical Depression	0.684	(0.523, 0.895)	**0.006**	0.672	(0.502, 0.900)	**0.008**
Dysphoric Mood	0.762	(0.609, 0.953)	**0.017**	0.769	(0.606, 0.975)	**0.030**
WAV	0.977	(0.868, 1.099)	0.693	0.965	(0.853, 1.093)	0.578
Anxiety	0.863	(0.698, 1.066)	0.171	0.881	(0.710, 1.093)	0.250
Hopelessness	0.738	(0.575, 0.946)	**0.017**	0.742	(0.570, 0.966)	**0.026**
Memory Complaint	0.901	(0.641, 1.267)	0.550	0.909	(0.645, 1.282)	0.587

Note: Model 1 adjusted for time to visit, age, education, sex, and Mini-Mental State Examination. Model 2 additionally adjusted for hemoglobin A_1c_ levels, duration of type 2 diabetes, body mass index, creatinine, total cholesterol, triglycerides, systolic and diastolic blood pressure.

* Mean Estimate: Negative Binomial Models are used to estimate the association between Ashkenazi and Total Geriatric Depression Scale Score, Dysphoric Mood, Withdrawal-Apathy-Lack of Vigor, and Hopelessness. Mean Estimate is interpreted as Ratios. Logistic Models are used to estimate association between Ashkenazi and Clinical Depression, Anxiety, and Memory Complaint. Mean Estimate is interpreted as Odds Ratios.

A two – sided *P* value of < 0.05 significance level is shown by boldface type.

## Data Availability

The data has not been previously presented orally or by poster at scientific meetings.

## Appendix A. Supporting information

Supplementary data associated with this article can be found in the online version at doi:10.1016/j.inpsyc.2025.100034.

## References

[1] Abd Ghafar MZA, Frailty and diabetes in older adults: overview of current controversies and challenges in clinical practice. Front Clin Diabetes Health 2022;3:895313. 10.3389/fcdhc.2022.895313. PMC10012063 36992729

[2] Abramson G Encyclopedia of Modern Jewish Culture 2004 Routledge London.

[3] Adams KB, Matto HC, Sanders S. Confirmatory factor analysis of the geriatric depression scale. Gerontologist 2004;44:818–26. 10.1093/geront/44.6.818. 15611218

[4] Beeri MS, The Israel Diabetes and Cognitive Decline (IDCD) study: design and baseline characteristics. Alzheimers Dement 2014;10:769–78. 10.1016/j.jalz.2014.06.002. 25150735 PMC4330555

[5] Blazer D, Hughes DC, George LK. The epidemiology of depression in an elderly community population. Gerontologist 1987;27:281–7. 10.1093/geront/27.3.281. 3609795

[6] Cohen Y, Haberfeld Y, Alon S, Heller O, Endeweld M (2023). Ethnic Gaps in Higher Education and Earnings Among Second and Third Generation Jews in Israel. https://www.ruppin.ac.il/media/blfi0s0k/%D7%92%D7%99%D7%9C%D7%99%D7%95%D7%9F-13-26-9-23-%D7%97%D7%93%D7%A9-%D7%90%D7%97%D7%A8%D7%99-%D7%9B%D7%9C-%D7%94%D7%AA%D7%99%D7%A7%D7%95%D7%A0%D7%99%D7%9D-%D7%9C%D7%A4%D7%A8%D7%A1%D7%95%D7%9D-%D7%91%D7%90%D7%AA%D7%A8.pdf (accessed May 17 2024).

[7] Daly M Prevalence of depression among adolescents in the U.S. from 2009 to 2019: analysis of trends by sex, race/ethnicity, and income. J Adolesc Health 2022;70:496–9. 10.1016/j.jadohealth.2021.08.026. 34663534

[8] Daoud N, Soskolne V, Mindell JS, Roth MA, Manor O. Ethnic inequalities in health between Arabs and Jews in Israel: the relative contribution of individual-level factors and the living environment. Int J Public Health 2018;63:313–23. 10.1007/s00038-017-1065-3. 29273838

[9] Dolberg P, Shiovitz-Ezra S, Ayalon L. Migration and changes in loneliness over a 4-year period: the case of older former Soviet Union immigrants in Israel. Eur J Ageing 2016;13:287–97. 10.1007/s10433-016-0391-2. 28804384 PMC5550612

[10] Fan Q, DuPont-Reyes MJ, Hossain MM, Chen LS, Lueck J, Ma P. Racial and ethnic differences in major depressive episode, severe role impairment, and mental health service utilization in U.S. adolescents. J Affect Disord 2022;306:190–9. 10.1016/j.jad.2022.03.015. 35301042

[11] Guerrero-Berroa E, Ethnicity/culture modulates the relationships of the haptoglobin (Hp) 1–1 phenotype with cognitive function in older individuals with type 2 diabetes. Int J Geriatr Psychiatry 2016;31:494–501. 10.1002/gps.4354. 26388309 PMC5753413

[12] Kaplan G, Depression among Arabs and Jews in Israel: a population-based study. Soc Psychiatry Psychiatr Epidemiol 2010;45:931–9. 10.1007/s00127-009-0142-1. 19777147

[13] Kashti O (2021). When it comes to education, Israel’s Ashkenazi-Mizrahi divide is still growing, Haaretz (June 11), n.p. https://www.haaretz.com/israel-news/2021-06-11/ty-article/.premium/when-it-comes-to-education-israels-ashkenazi-mizrahi-divide-is-still-growing/0000017f-f4cd-ddde-abff-fced39a60000 (accessed May 17 2024).

[14] Kirmayer LJ. Cultural variations in the clinical presentation of depression and anxiety: implications for diagnosis and treatment. J Clin Psychiatry 2001;62(13):22–8. discussion 29–30. 11434415

[15] Kohn R, Levav I. Jews and their intraethnic differential vulnerability to affective disorders, fact or artifact? I: an overview of the literature. Isr J Psychiatry Relat Sci 1994;31:261–70. 7875950

[16] Kohn R, Levav I, Dohrenwend BP, Shrout PE, Skodol AE. Jews and their intraethnic vulnerability to affective disorders, fact or artifact? II: evidence from a cohort study. Isr J Psychiatry Relat Sci 1997;34:149–56. 9231577

[17] Lavie I, Decrease in gait speed over time is associated with increase in number of depression symptoms in older adults with type 2 diabetes. J Gerontol A Biol Sci Med Sci 2023;78:1504–12. 10.1093/gerona/glad008. 36626301 PMC10395563

[18] Marwijk HW, Wallace P, de Bock GH, Hermans J, Kaptein AA, Mulder JD. Evaluation of the feasibility, reliability and diagnostic value of shortened versions of the Geriatric Depression Scale. Br J Gen Pract 1995;45:195–9. 7612321 PMC1239201

[19] Mayeda ER, Glymour MM, Quesenberry CP, Whitmer RA. Inequalities in dementia incidence between six racial and ethnic groups over 14 years. Alzheimers Dement 2016;12:216–24. 10.1016/j.jalz.2015.12.007. 26874595 PMC4969071

[20] Mayeda ER, Karter AJ, Huang ES, Moffet HH, Haan MN, Whitmer RA. Racial/ethnic differences in dementia risk among older type 2 diabetic patients: the diabetes and aging study. Diabetes Care 2014;37:1009–15. 10.2337/dc13-0215. 24271192 PMC3964496

[21] Montorio I, Izal M. The Geriatric Depression Scale: a review of its development and utility. Int Psychogeriatr 1996;8:103–12. 10.1017/s1041610296002505. 8805091

[22] Moulton CD, Pickup JC, Ismail K. The link between depression and diabetes: the search for shared mechanisms. Lancet Diabetes Endocrinol 2015;3:461–71. 10.1016/s2213-8587(15)00134-5. 25995124

[23] Netz Y, Goldsmith R, Shimony T, Arnon M, Zeev A. Loneliness is associated with an increased risk of sedentary life in older Israelis. Aging Ment Health 2013;17:40–7. 10.1080/13607863.2012.715140. 22913477

[24] Parmelee P, Lawton M, Katz I. Psychometric properties of the Geriatric Depression Scale among the institutionalized aged. Psychol Assess 1989;1:331–8. 1, 331–338.

[25] Pickett YR, Bazelais KN, Bruce ML. Late-life depression in older African Americans: a comprehensive review of epidemiological and clinical data. Int J Geriatr Psychiatry 2013;28:903–13. 10.1002/gps.3908. 23225736 PMC3674152

[26] Pratt L, Brody D. Depression in the US Household Population, 2009–2012. US Govt. Hyattsville, MD: Printing Office,; 2014. 25470183

[27] Ravona-Springer R, Increase in number of depression symptoms over time is related to worse cognitive outcomes in older adults with type 2 diabetes. Am J Geriatr Psychiatry 2021;29:1–11. 10.1016/j.jagp.2020.09.022. 33127316 PMC7771631

[28] Ruskin PE, Depressive symptoms among community-dwelling oldest-old residents in Israel. Am J Geriatr Psychiatry 1996;4:208–17. 10.1097/00019442-199622430-00004. 28531079

[29] Schwartz E, Khalaila R, Litwin H. Contact frequency and cognitive health among older adults in Israel. Aging Ment Health 2019;23:1008–16. 10.1080/13607863.2018.1459472. 29723058 PMC6215531

[30] Sheikh JI, Yesavage JA. Geriatric depression scale (GDS): recent evidence and development of a shorter version. Clin Gerontol 1986;5:165–73.

[31] Shoji T, Association between comorbidities associated with diabetes and higher-level functional status in older patients with type 2 diabetes mellitus: a cross sectional study. Eur Geriatr Med 2024. 10.1007/s41999-024-00937-8. PMC11377677 38340284

[32] Simhi M, Schiff M, Pat-Horenczyk R. Economic disadvantage and depressive symptoms among Arab and Jewish women in Israel: the role of social support and formal services. Ethn Health 2024;29:220–38. 10.1080/13557858.2023.2279479. 37938146

[33] Soleimani L, Specific dimensions of depression have different associations with cognitive decline in older adults with type 2 diabetes. Diabetes Care 2021;44:655–62. 10.2337/dc20-2031. 33468519 PMC7896256

[34] Soleimani L, Schnaider Beeri M, Grossman H, Sano M, Zhu CW. Specific depression dimensions are associated with a faster rate of cognitive decline in older adults. Alzheimers Dement (Amst 2022;14:e12268. 10.1002/dad2.12268. 35317432 PMC8923346

[35] Turner RJ. Understanding Health Disparities: The Promise of the stress Process Model. New York: Springer,; 2009.

[36] USDHHS. Mental Health: Culture, reace, and ethnicity—A supplement to mental health: A report of the Surgeon General. Rocville, MD: US Govt. Printing Office,; 2001. 20669516

[37] Yang TC, Park K. Racial/ethnic disparities in depression: investigating how sources of support and types of integration matter. Soc Sci Res 2019;82:59–71. 10.1016/j.ssresearch.2019.04.002. 31300084

[38] Anxiety Zisberg A. and depression in older patients: the role of culture and acculturation. Int J Equity Health 2017;16:177. 10.1186/s12939-017-0666-z. 28978328 PMC5628440

