# Supplementary material for: Ethnicity predicts long-term depressive symptom patterns in older adults with type 2 diabetes
Source: Int Psychogeriatr. Author manuscript; Available in PMC 2026 May 22. (PMC13196895; doi:10.1016/j.inpsyc.2025.100034)
Supplement: 1 [file NIHMS2170172-supplement-1.docx]

Supplemental Table 1. Demographic and clinical characteristics of the dropout participants after baseline.

|  | **Dropout after**  **Baseline (N=151)** | **At least One**  **Follow-up(s) (N=751)** | **P-value** | **Total (N=902)** |
| --- | --- | --- | --- | --- |
| **Age (years)** |  |  |  |  |
| Mean (SD) | 72.9 (4.75) | 72.1 (4.65) | 0.07 | 72.3 (4.67) |
| **Female sex (%)** | 64 (42.4%) | 293 (39.0%) | 0.440 | 357 (39.6%) |
| **Years of education** |  |  |  |  |
| Mean (SD) | 12.8 (3.55) | 13.2 (3.51) | 0.152 | 13.1 (3.52) |
| **Duration of T2D (years)** |  |  |  |  |
| Mean (SD) | 10.2 (4.27) | 9.63 (4.40) | 0.189 | 9.72 (4.38) |
| **Cholesterol mg/dl** |  |  |  |  |
| Mean (SD) | 175 (23.3) | 173 (24.8) | 0.486 | 174 (24.5) |
| **Creatinine mg/dl** |  |  |  |  |
| Mean (SD) | 1.06 (0.47) | 1.01 (0.29) | 0.182 | 1.01 (0.328) |
| **HbA1c (%)** |  |  |  |  |
| Mean (SD) | 6.91 (0.76) | 6.82 (0.76) | 0.214 | 6.84 (0.757) |
| **Triglycerides mg/dl** |  |  |  |  |
| Mean (SD) | 154 (54.8) | 156 (63.2) | 0.771 | 156 (61.8) |
| **Systolic blood pressure (mmHg)** |  |  |  |  |
| Mean (SD) | 135 (9.14) | 134 (8.50) | 0.089 | 135 (8.62) |
| **Diastolic blood pressure (mmHg)** |  |  |  |  |
| Mean (SD) | 75.1 (4.49) | 75.6 (4.61) | 0.199 | 75.7 (4.67) |
| **BMI (Kg/m2)** |  |  |  |  |
| Mean (SD) | 28.8 (4.72) | 28.6 (4.10) | 0.622 | 28.6 (4.21) |
| **MMSETotal** |  |  |  |  |
| Mean (SD) | 27.7 (1.97) | 28.1 (1.74) | **0.027** | 28.0 (1.79) |
| **Clinical Depression** | 25 (15.1%) | 113 (16.6%) | 0.221 | 138 (15.3%) |
| **Total GDS** |  |  |  |  |
| Mean (SD) | 2.46 (2.51) | 2.20 (2.34) | 0.220 | 28.0 (1.79) |

Note: MMSE = Mini-Mental State Examination.
